# Risk Factors for African Swine Fever in Wild Boar in Russia: Application of Regression for Classification Algorithms

**DOI:** 10.3390/ani15040510

**Published:** 2025-02-11

**Authors:** Olga I. Zakharova, Elena A. Liskova

**Affiliations:** Federal Research Center for Virology and Microbiology, Branch in Nizhny Novgorod, 603950 Nizhny Novgorod, Russia; liskovaea@mail.ru

**Keywords:** African swine fever, population density, risk factors, carcasses, logistic regression, random forest model, wild boar, Russian Federation

## Abstract

The population density of susceptible animals, including domestic pigs and wild boar, is a major risk factor for the emergence of African Swine Fever outbreaks. The ASF foci in wild boar in Russia is sustained by the presence of the virus in the environment, which is primarily determined by the number of infected carcasses found. This study investigates the risk factors related to the occurrence of ASF virus among wild boar, identified through passive monitoring and depopulation control measures, by employing generalized logistic regression models and random forest analysis. The random forest regression outperformed logistic regression coefficient of determination (R^2^ = 0.98 and R^2^ = 0.88) according to the statistical modeling of ASF using different regression types. When comparing regression models, the results showed that wild boar population density, the number of hunting farms, the presence of infected carcasses, and ASF outbreaks among domestic pigs were the main predictors of epidemic in wild boar. The application of a multiple logistic regression model confirmed the significance of the identified risk factors, determining of the probability of ASF outbreaks among wild boar. Given the prolonged affected area across most regions of Russia, the random forest model proved to be the most effective and interpretable based on quality indicator assessments. By highlighting the important role of geographical conditions, identifying these risk factors enhances our understanding of ASF dynamics in specific regions and offers valuable information for decision-makers in developing targeted control strategies against this disease.

## Simple Summary

The prolonged African swine fever (ASF)-affected areas of Russia necessitate understanding the regional features of the spread of the epidemic, as geographical conditions play a crucial role in spreading disease. The results of the study underline the importance of predictive modeling to highlight the complex interplay between wildlife, domestic pigs, and ecology factors in the epidemic process. Utilizing predictive models through classification and regression algorithms enhances our understanding of the epidemiology of the disease. By identifying key risk factors such as wild boar density, the presence of infected carcasses, and interactions with domestic pigs, these models provide insights that inform targeted interventions. The study insights aim to guide decision-makers in devising effective control strategies to mitigate the spread of disease, ultimately contributing to better management of this emergence. Implementing advanced statistical techniques, like random forest analysis, allows for improved prediction accuracy, which is essential for developing strategic control measures.

## 1. Introduction

African swine fever (ASF) is a viral disease that poses a health risk to all members of the Suidae and causes enormous damage to the pig industry in many countries [1]. The management of the epidemic that influences the factors of ASF transmission among wild boar and domestic pigs can help in developing effective strategies to combat the disease and minimize the risk of further expansion of the virus into safe areas [2]. The spread of ASF is a complex epizootic process that involves several routes of virus transmission. The primary mode of transmission occurs through direct contact between infected and healthy animals, when the pathogen is transferred from one animal to another [3]. In large pig farms, a rapid and mass transmission of infection is facilitated by the high concentration of domestic animals in a congested population [4,5,6].

It is important to recognize that the indirect pathway of transmission of the ASF virus plays an equally important part in establishing affected territories. Infected carcasses of wild boar retain the virus and represent a source of infection for healthy populations. They also act as a reservoir of infection by contaminating environmental factors. This contamination creates direct conditions for the further spread of infection in wildlife [7,8,9].

An important part of the relationship between infected wild boar and the environment has been revealed, and this has been important in the current ASF epidemic in European countries. This relationship is critical for understanding the mechanism of ASF virus transmission between domestic swine and wild boar populations. Climate, biotic conditions, environmental factors, and the environment of wild boar habitats can play a crucial role in determining the spread of ASF foci. Wild boar moving through their habitat may come into contact with various sources of infection, including food residues, other susceptible animals, or their remains [5,10,11].

Passive monitoring, which involves searching for the carcasses or remains of wild boar, plays a crucial role in epidemic ASF control measures. It helps prevent the further spread of the disease and protects both wild and domestic pigs. To create an effective passive monitoring system for wildlife, regular patrols of forested and agricultural areas are conducted, along with joint operations involving veterinary services and the Ministry of Natural Resources in areas where wild boar habitats are likely to be found in Russia.

Specially trained teams of specialists search for wild boar carcasses and remains. A significant increase in wildlife monitoring is associated with modern technologies such as drones and video surveillance cameras (photo and video traps). This approach allows large areas to be covered and speeds up the process of detecting boar, facilitating the effective implementation of preventive measures [12,13,14].

Risk assessment (RA) is a systematic and comprehensive process focused on collecting, analyzing, and documenting information about potential threats. This procedure is essential for establishing appropriate risk levels in various situations, allowing for informed decision-making aimed at mitigating adverse effects. Additionally, examining spatial patterns of risk has gained importance in the field of risk assessment. By mapping risk factors geographically, one can identify areas that are particularly vulnerable to foci and health emergencies in animals [15,16].

A study of data from multiple publications indicates that risk factors for the African swine fever (ASF) virus spread among wild boar include many predictors and differ with epidemic scenarios.

Changes in habitat conditions, interactions between other domestic pigs, wild boar population density, movement of live animals and pig products, and the transmission of viruses through fomites are among the factors that may influence this epidemic process [14,15,16].

Statistical modeling is a valuable tool in epidemiology that is applied in various tasks of factor analysis to identify the dependencies of risk factors for each geographically marked area, taking into account regional conditions. Local spatial variation in risk factors relevant to ASF foci in domestic pigs depends on the type of pig husbandry and biosecurity measures in place, while the risk of introduction of pathogens in wild boar is related to natural habitat factors [16,17,18].

To predict and control the further spread of ASF beyond the existing geographical range of wild boar, predictive models are employed that link the locations of registered cases with environmental factors [17,18]. For example, Cappai et al. (2018) used a negative binomial regression model to identify climatic and socioeconomic factors in assessing the risk of ASF in Sardinia [19]. In addition, the results of our previous studies, which modeled the dependence of ASF outbreaks in domestic pigs and wild boar on several risk factors using logistic and negative binomial regression models, identified significant predictors [13,18,20].

The high importance of the issue concerning ASF virus transmission and the risk factors for infection spread among both wild boar and domestic pigs [21,22] influenced our choice of research focus.

The aim of this work was to compare two regression models, logistic and random forest, in order to identify ASF risk factors among wild boar and to compare their predictive performance in the probability of occurrence of disease.

## 2. Materials and Methods

### 2.1. Study Area

The analysis was performed on a dataset concerning ASF outbreaks among wild boar in affected subjects of the Russian Federation (RF). Included in the statistical processing were only those areas where the disease was regularly observed during the analysis period. Information on wild boar population density was requested and obtained from regional nature ministries. A study of affected territories in Russia resulted in the analysis of two oblasts: the European part, which comprised 34 regions, and the Far Eastern part, consisting of 4 regions and a total of 2490 municipal entities (districts).

### 2.2. Dataset

**Figure 1:**
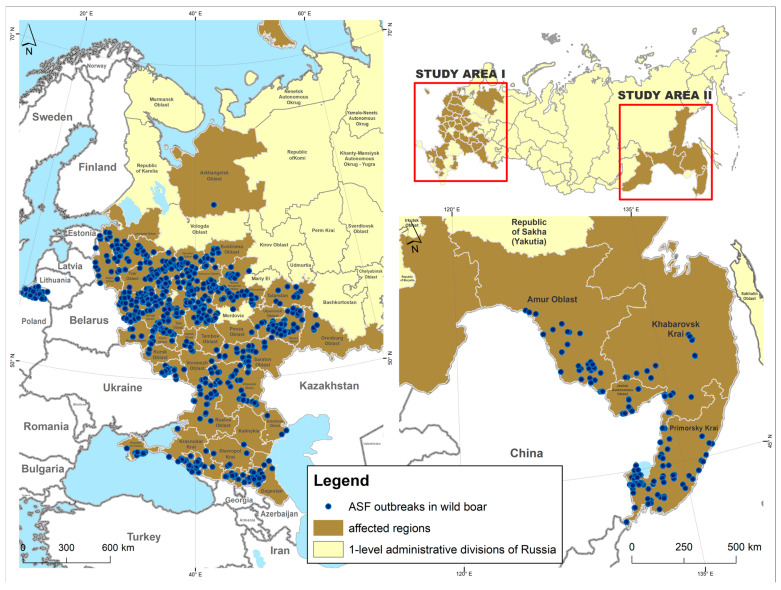
The African swine fever outbreaks in wild boar of Russia from 2007 to 2023.

Information on the occurrence of ASF in Russia for the period from 2007 to 2023 was extracted from the database of the World Organization for Animal Health’s (WOAH) [23]. In total, 1139 outbreaks of ASF wild boar were registered in the affected areas during the analyzed period, of which 982 were in the European part and 157 in the Far Eastern part of Russia. In this study, we considered an ASF “outbreak” as a laboratory-confirmed and WOAH-notified event in an individual boar, defined by geographic coordinates and date of occurrence [23] (Figure 1).

### 2.3. Explanatory Predictors

The most significant factors playing a role in the spread of ASF among wild boar were selected as independent variables, according to the analysis of literary data [17,22,24].

Data on the number of wild boar in the study areas in dynamics from 2007 to 2023, as well as information on animals found dead from ASF and their carcasses, were obtained from statistical reports of the regional ministries of natural resources and ecology of the Russian Federation (https://www.mnr.gov.ru/about/, accessed on 10 November 2024). The assessment of wild boar population density was carried out based on the data obtained from the number of boar censuses requested, which were conducted in designated hunting areas that include forestry areas of districts by the regional ministries of nature.

The landscape variables of the affected areas were collected from vector and raster GIS layers of the Open Street Map (OSM) (https://www.openstreetmap.org/#map=3/69.62/-74.90, accessed on 12 November 2024). The list of landscape-climatic variables included the percentage of water bodies (rivers and lakes), area and proportion of vegetation cover, and length and density of roads. All variables were extracted and summarized by district, with median values calculated using GIS zonal statistics tools. Data on population density and settlements in the district were obtained from the website of the Federal State Statistics Service (https://rosstat.gov.ru/, accessed on 12 November 2024). Table 1 shows the explanatory predictors used in the classification algorithms.

Variables were also examined for mutual correlation through the Variance Inflation Factor statistical test to prevent multicollinearity [22]. In each pair of correlated variables, the variable with the lowest coefficient correlation was selected for analysis.

### 2.4. Study Design

Regression models were used to investigate the relationships between the presence of ASF outbreaks in each district and various predictors. We evaluated two models: a logistic model, where the response variable was the binary or presence of outbreaks in the areas, and a random forest model. Multiple explanatory predictors listed in Table 1 were included in both models.

Regression modeling was carried out simultaneously for two geographic areas, conditionally divided into the European part and the Far Eastern part of the Russian Federation.

#### 2.4.1. Preprocessing

The response variable contained a total of 3468 rows of dataset, of which 1139 represented the presence of ASF outbreaks in the subject areas of the region. Although using such data to train machine learning models can lead to high accuracy, other performance metrics, like precision and recall, are often insufficient.

If this imbalance in the data is not properly addressed, the results may be inaccurate and the predictions will not be effective. Therefore, to develop an effective model, it is essential to first address this imbalance in the data. For this purpose, the SMOTE technique was used in R version 4.4.1 (26 September 2024) [25,26].

The next stage is to construct the model after completing data preparation and addressing the imbalanced dataset. To enhance the accuracy and efficiency of this process, the dataset is divided with an 80% training data and 20% testing data ratio. After the split, the model is trained using various classification methods, specifically random forest and logistic regression as the algorithms employed in this study.

#### 2.4.2. General Logistic Regression Model

Logistic regression is a statistical method used for binary classification algorithm, where the goal is to predict the probability of a certain event occurring based on categorical predictors. The logistic function maps any real-valued number into a value between 0 and 1 [27].

The results from logistic regression allow for the calculation of odds ratios, which are often used to express the strength of the association between the predictor variables and the outcome. This is particularly useful for interpreting the impact of risk factors.

The response predictor is the occurrence of ASF in the wild boar population in the area during the year [28,29]. Various socioeconomic and environmental explanatory factors were included in the analysis, as detailed in Table 1. To eliminate redundant variables, a preliminary assessment of the Variance Inflation Factor (VIF) was conducted. A threshold of 5 was used, resulting in the exclusion of all variables with a VIF greater than 5 from further modeling [30].

After training, the model’s performance can be evaluated using various metrics, such as accuracy, precision, recall, and the area under the ROC curve (AUC-ROC), which helps assess how well the model distinguishes between the two classes. To assess the model’s goodness of fit, the Hosmer–Lemeshow test was used to examine the significance of differences between expected and observed proportions. Moran’s I spatial autocorrelation test was conducted to analyze the spatial distribution of the response variable and the model’s residuals, evaluating how closely the observed spatial distribution matches a random distribution [28].

#### 2.4.3. Random Forest Model

The random forest classification method was employed to explore the connections between the categorical factor of ASF outbreak presence and a variety of potential explanatory predictors [31,32]. This technique is a supervised machine learning approach that leverages an ensemble of decision trees created from observed values and variables to perform classifications (for categorical variables) or regressions (for numerical variables) [33]. The method involves constructing numerous decision trees, each generated from a bootstrap sample of the original training dataset [33]. The regression estimates are obtained by averaging the regression results of all individual trees, while the final model is determined by majority voting.

One of the most attractive aspects of random forest is its flexibility. It can be applied to both random forest and regression tasks, and it clearly indicates the overall importance assigned to the input features [31].

In our study, we trained the model using 1000 decision trees, randomly selecting variables for each tree, and employed another 1000 trees for validation, with 25% of the input data chosen randomly for this purpose.

Additionally, the model provides metrics reflecting the importance of the explanatory variables, offering “importance” and “percentage” values. The “importance” score is calculated based on the sum of all Gini coefficients, which can be interpreted as the frequency with which a variable contributes to a split, weighted by the significance of that split relative to the total number of decision trees. Conversely, the “percentage” indicates the proportion of a particular variable’s Gini coefficients in relation to the total sum of Gini coefficients [32].

The quality of the regression model was evaluated using the coefficient of determination (R^2^), ROC (receiver operating characteristic curve) curve, and Accuracy in Confusion Matrix. The statistical metrics of the performance of the model are presented for (1) the training data and (2) the validation data testing prediction [34,35].

#### 2.4.4. Evaluation of Performance of the Models in a Comparative Aspect

In order to measure the performance of the proposed classification algorithms, a number of evaluation metrics have been introduced, including sensitivity, specificity, accuracy. Other measures include the ROC curve and R^2^.

1. R-squared (R^2^) (coefficient determination) represents the square of the correlation between the observed values of the outcome and the predicted values of the model. The higher the adjusted R^2^, the better the model.
2. ROC (receiver operating characteristic curve) Curve and AUC (Area Under the Curve) Score. The AUC of the ROC curve serves as a comprehensive metric that evaluates the overall performance of the model across all possible thresholds. The performance of the final regression models for the European part and Far East part of Russia was estimated based on the predictive powers of the candidate models. Model performance was evaluated using the area under the receiver operating characteristic curve (AUC), which plots model sensitivity (true positive rate—TPR) against specificity (false positive rate—FPR) [36]. AUC values under 0.7 indicate relatively poor accuracy, while values above 0.7 are considered acceptable. In this study, models with an AUC greater than 0.7 were deemed accurate. The AUC measures the overall effectiveness of the binary classification model, with values ranging from 0 to 1; a higher AUC reflects better model performance [34].
3. In the context here, sensitivity and specificity reflect the ability of the model to predict affected and non-affected areas of ASF foci in wild boar, respectively. In addition to the true validation, the random forest and logistic regression models were cross validated using k-fold cross validation (k = 10). Cross-validation is a process of excluding a set of ASF boar-infected locations from model fitting. The probability of finding a focus at these omitted locations is then estimated from the fitted model output [37].

### 2.5. Software

Initial data processing and evaluation were performed using Microsoft Office Excel program (Microsoft Corporation, Redmond, WA, USA). Visualization of the ASF outbreaks in wild boar population size was conducted using ArcMap version 10.8.2 (Esri, Redlands, CA, USA). The regression algorithms were carried out in the software environment R.

## 3. Results

### 3.1. Descriptive Analysis

**Figure 2:**
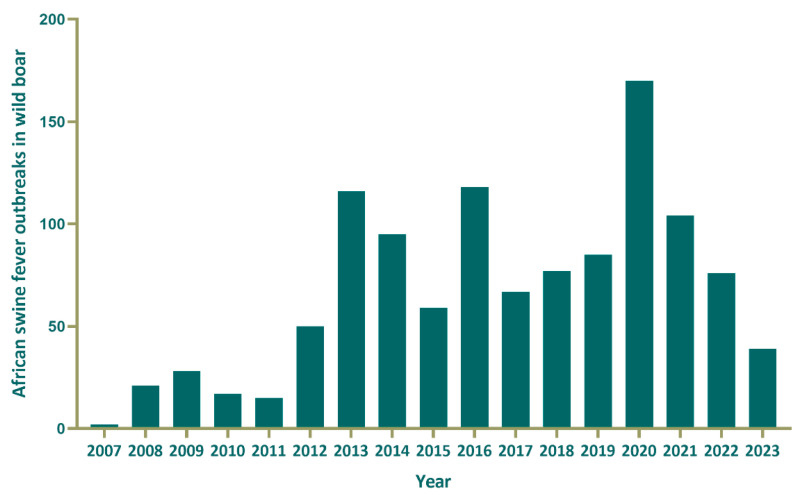
Dynamics of ASF outbreaks among wild boar in Russia from 2007 to 2023.

The incidence rate of ASF outbreaks in the wild population in Russia accounts for 41.7% of the total number of foci during the observed period, underscoring the extent of the virus spread. The most pronounced peaks in the ASF foci were observed in 2013 (116 outbreaks), 2016 (118 outbreaks), 2020 (170 outbreaks), and 2021 (104 outbreaks). Geographically, it is focused in specific regions of Russia. Researchers noted the highest incidence of ASF in wild boar in Ryazan, Moscow, Tula, Tver, and Vladimir Oblasts. ASF outbreaks were also registered in Smolensk, Samara, and the bordering Pskov and Leningrad regions. The Far Eastern region has endured endemic areas of disease for many years, especially in the border of Primorsky Krai and Amur Oblast. These areas maintain segments with high wild boar population densities, creating favorable conditions for the maintenance and spread of the virus (Figure 2).

The spatial and temporal peculiarities of the infection manifestation in different regions are noted, characterized by stationary disease presence with periodic ASF outbreaks in certain territories and a tendency to persist in the wildlife. Additionally, there are short-term but large-scale epidemics with widespread coverage, including areas that are currently unaffected in Russia.

### 3.2. Regression Models for Classification Algorithm

#### 3.2.1. Logistic Regression Models

The general logistic regression model, which defines the relationship between the occurrence of ASF outbreaks and risk factors, had a coefficient of determination of 0.65. The general logistic regression model presented the following key predictors that play an important role in the occurrence of ASF foci in the European part of Russia: the wild boar population density, the carcasses wild boar, and the number of ASF outbreaks among domestic pigs (Table 2).

Breeding and monitoring indicators of wild boar in the Far East based on the number hunting farms of demonstrated a high significance in the probability of detecting ASF outbreaks in the regions of problem areas in the Far East. Additionally, the regression model identified the wild boar population density, number of carcasses, and number of settlements as key predictors of ASF. More factors that have statistical significance in detecting ASF foci in wild boar in the Far East part of Russia are presented in Table 3.

#### 3.2.2. Random Forest Model in Evaluation Risk Factors of ASF in Wild Boar

**Figure 3:**
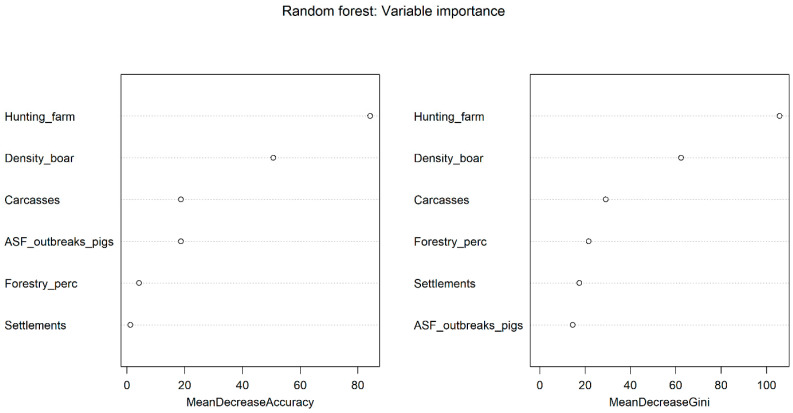
Variable importance of the random forest model of ASF outbreaks in wild boar in the European part of Russia.

The results obtained during the analysis of the random forest classification and regression algorithm in assessing the significance of variables influencing the occurrence of ASF outbreaks among wild boar in the European part of Russian are shown in Figure 3.

Training of the model based on the random forest using data on ASF incidence among wild boar in the European part of Russia showed a high degree of fit of the model (R^2^ = 0.80; *p* ≤ 0.001) and a good degree of fit to the cross-validation dataset (R^2^ = 0.78; *p* ≤ 0.001).

The relative significances of the factor modelling results are shown in Table 4. The results of the random forest demonstrated that socio-demographic factors (number of hunting farms, wild boar population density, number of carcasses and remains) were most important in explaining the observed distribution of ASFV in the European part of Russia, while the role of landscape factors such as forestry areas and number of settlements was less significant. The test of regression residuals for spatial autocorrelation showed no tendency for clustering of residuals (Moran’s I = −0.023; z-score = −0.327; *p* ≤ 0.632).

**Figure 4:**
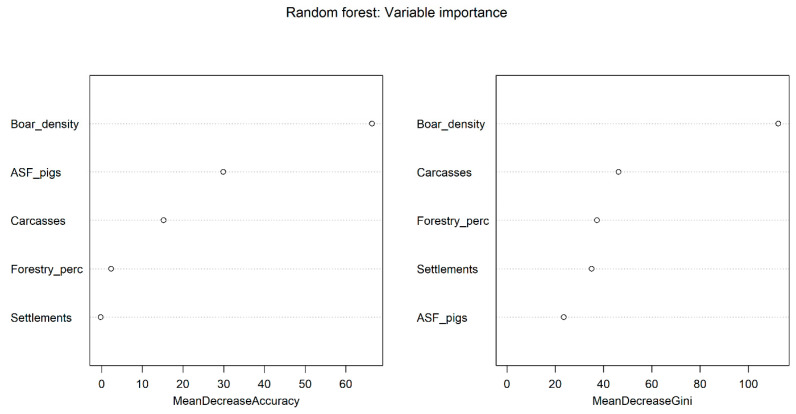
Variable importance of random forest model of ASF outbreaks in wild boar in the Far East part of Russia.

The results obtained during the analysis of the random forest model for assessing the significance of variables influencing the occurrence of ASF outbreaks among wild boar in the Far East part of the Russian Federation are presented in Figure 4.

Training of the model based on the random forest using data on ASF among wild boar in the Far East part of Russia also showed a good degree of fit of the model to the training data (R^2^ = 0.75; *p* ≤ 0.001) and a good degree of fit to the cross-validation dataset (R^2^ = 0.74; *p* ≤ 0.001).

The relative significance of the variables modelling results is presented in Table 5. The results of the random forest classification algorithm demonstrated that socio-demographic factors (wild boar population density, number of carcasses) were most important in explaining the observed distribution of the outbreaks in the Far East part of Russia, while the role of landscape factors such as ASF outbreaks in domestic pigs and number of settlements was less significant. The test of regression residuals for spatial autocorrelation showed no tendency for clustering of residuals (Moran’s I = −0.025; z-score = −0.342; *p* ≤ 0.432).

#### 3.2.3. Comparative Assessment of the Predictive Performance of Regression Models

The characteristics of the quality assessment indicators for the fit of the logistic and random forest models, applied for analyzing the risk factors of ASF outbreaks among wild boar in the European part and the Far Eastern part of the Russian Federation region, are demonstrated in Table 6.

From Table 6, it is clear that the random forest and logistic regression algorithms have an acceptable level of accuracy, but the random forest algorithm is a preferable option because of its higher level of accuracy. The level of accuracy in the random forest model for the ASF training data in the Far East has the highest value of 0.98, compared to the European part of Russia, but remains quite good. The logistic regression model gave the lowest level of accuracy but it is still acceptable for a regression model.

The determination indicators demonstrated values characterizing good and sufficient predictive ability of the models. At the same time, the random forest classification and regression model was the best model, with a determination indicator for test data of 0.80–0.78 for the European part and 0.75–0.74 for the Far East part of Russia against 0.67–0.65 for the European part and 0.70–0.68 for the Far East part of Russia, characterizing the logistic regression model.

The area under the ROC curve, determining the excellent quality of the constructed model, gave values of 0.94 and 0.81 for the random forest classification model for the validation dataset. The logistic regression model had satisfactory AUC values, which were in the range from 0.78 to 0.75 for the validation dataset of ASF outbreaks in Russia.

## 4. Discussion

Analyzing the risk factors contributing of African swine fever (ASF) in wild boar represents an important area of research in determining the choice of effective control measures. Identifying the factors that contribute to ASF outbreaks in wild boar is critical to developing strategies to prevent the virus from entering unaffected areas [38,39,40].

Research on ASF virus transmission variables in domestic pig and wild boar populations is ongoing to understand how and by what processes the virus survives outside the animal and what factors contribute to its spread [5,10,18].

The risk factors for African Swine Fever foci in wild boar may include demographic variable, climatic conditions, the presence of natural virus carriers, various types of hunting, and items involved in hunting activities [5,18,41]. Additionally, the behavior and migratory activity of wild boar, as well as their interaction with domestic pigs, which can serve as additional targets for the spread of the infection, are also important factors [42,43]. Environmental factors that directly influence the probability of finding diseased wild boars determine the spatial and temporal patterns of ASF spread in wild populations. In terms of optimal wild boar habitats, any type of land cover that provides shelter, water, and food for animals should be considered in the model as an ecological risk factor for ASF occurrence [44,45].

The importance of assessing the current epidemic necessitates various forecasting methods to evaluate the risk of further disease spread among wild boar in unaffected areas.

The aim of this work was to assess the performance metrics of models and identification of risk factors using algorithms for classification and regression in order to modeling ASF outbreaks in wild boar in Russia. As a results of statistical modeling, we determined that the random forest classification and regression model was the best for forecasting. By applying these types of regression, we identified the factors that were most significant in the likelihood of ASF outbreaks in wild boar across regions of the Russian Federation, which vary in geographical and area conditions.

A limitation of this research by evaluation of quality in models was the incompleteness of data on wild boar population density in Russia due to the lack of data covering geographical areas within wild boar habitats.

The analysis of the training and testing datasets of the statistical models indicates the optimal selection of variables with predictive power for the performed analysis with the random forest algorithm. The ROC curve of the model in this case for test data of ASF outbreaks in the European part of Russia was 0.98, while that for the Far Eastern region was 0.95. The AUC scores for the logistic regression model were 0.87 and 0.85, respectively.

The risk factors for the spread of ASF outbreaks among wild boar, as assessed by the logistic regression model, were similar for both the European part of Russia and the regions of the Far East. The main factors significant for ASF outbreaks among wild boar in the European part of Russia was the population density (OR = 1.26, [27.98–130.37], *p* ≤ 0.000), while in the territory of the subjects of the Far East, the number of hunting grounds was more significant (OR = 57.05, [3.845–185.345], *p* ≤ 0.000). An equally important factor in the spread of ASF in the subjects in Russia was the factor associated with the collection and search for carcasses of wild boar, the OR of which was 1.72 (1.56–5.32), *p* ≤ 0.000 in the European part and 1.38 (1.237–14.575), *p* ≤ 0.000 in the Far East in Russia.

According to the literature, carcasses of dead wild boar that have been left in environmental conditions for an extended period play a significant role in the preservation and transmission of the virus among wild boar, particularly in areas where ASF is endemic [8,46,47].

The important risk factors also included a predictor indicating the presence of ASF infection among domestic pigs in the region (OR = 1.07 [1.03–4.11], *p* ≤ 0.000). Consequently, the probability of an ASF outbreak among wild boar in the European part of Russia depends on the existing outbreaks among domestic pigs [9,18,48].

According to the results of the logistic regression and random forest, the risk factors for ASF in the wild boar population are primarily associated with the susceptible population. Additionally, these risk factors are associated with the number of farms engaged in breeding and keeping animals.

Uncertainty in assessing the number of boar in districts of Russia that are unfavorable for ASF can arise from the methods used for accounting and the environmental characteristics of this species. This uncertainty can lead to errors in determining the significance of wild boar populations as a significant factor in the spread of ASF within regions of Russia [49,50,51].

The clustering of susceptible animals, such as wild boar, plays a crucial role in the transmission chain of infection. Despite the potential significance of population density of wild boar as a risk factor for the spread of ASF, determining the actual population size and conducting constant monitoring is extremely difficult and practically unfeasible because of the animals’ continuous migrations [42,52,53].

It is important to consider that actual estimates of wild boar population density are likely rough approximations of the true absolute value and may be biased based on initial data.

The analysis of the comparison table of models by performance indicators presented the conclusion that the most effective of the applied models was the random forest model. In addition to a higher coefficient of determination from 0.8 to 0.7, the presented model has an area under the AUC curve equal to 0.94 according to the European data and 0.78 according to the Far East data. Therefore, it has greater accuracy and better responsiveness and correctness. It is also evident from the table that all classification algorithms have an acceptable level of accuracy, but the random forest algorithm is the preferred option due to its higher level of accuracy. In this study, when using the random forest model on the European part data, an accuracy of 94 percent was achieved, while the accuracy of the random forest regression model was 88 percent.

When comparing the coefficient of determination of the regression models, it was found that the random forest model had the best predictive power regarding the introduction of ASF foci among wild boar in the affected subjects of Russia. This model also proved to be the most effective at explaining the predicted probability of ASF outbreaks.

## 5. Conclusions

ASF is a contagious disease of pigs and wild boar that should be detected as early as possible to prevent further spread and the development of a new epidemic. Developing machine learning models can help in the early detection of ASF outbreaks in wild boar and subsequent prevention of its spread. This study examines the performance of several regression models in predicting ASF based on a number of variables that constitute risk factors for the spread of the disease. Given the prolonged ASF affected area across most regions in Russia, the random forest model proved to be the most effective and interpretable based on quality metrics assessments. Random forest classification outperformed the logistic regression model, with a classification accuracy of 98 percent. By highlighting the important role of geographical conditions, identifying risk factors enhances our understanding of ASF dynamics in specific regions, offering valuable information for decision-makers in developing targeted control strategies against this disease. Additionally, regression modeling can provide a basis for decision-making regarding the choice of preventive measures, including animal population management. Conducting regular raids to search for dead carcasses and remains of wild boar in unfavorable regions of the Russian Federation with the involvement of veterinary service specialists and regional ministries of nature for the protection of hunting resources will help to approach the issue of ASF foci eradication in wildlife.

## Figures and Tables

**Table 1:** Statistical metrics of significant variables of ASF spread among wild boar for affected districts of Russia.

Explanatory Predictors	Unit	Type Variables	Median (Minimum–Maximum)	VIF
Forestry areas	%	continuous	53.54 (1.60–77.2)	1.342
Water bodies	%	continuous	42.32 (0.23–88.32)	2.654
Vegetation areas	thousand km^2^	continuous	4065.56 (1400–154,000)	1.032
Human population density	persons/km^2^	continuous	8.53 (0.65–1234.6)	1.912
Settlements	unit	continuous	46 (8–1534)	1.925
ASF-infected boar carcasses	unit	continuous	2 (1–125.34)	1.978
Density of main roads	km/km^2^	continuous	48.8 (1.34–1254.32)	1.002
Percentage of hunting farms in the area	%	continuous	25 (1.54–1832.32)	3.283
Total area of hunting areas	km^2^	continuous	35.54 (10.23–1542.23)	3.326
ASF outbreaks in domestic pigs	unit (1 and 0)	categorical	15 (1–67)	2.435
ASF outbreaks in wild boar	unit (1 and 0)	categorical	3 (0–8)	1.231
Wild boar population density	head/km^2^	continuous	0.32 (0.008–3.321)	1.546

**Table 2:** Statistical metrics of the general logistic regression model used to determine risk factors of ASFV among wild boar populations in the European part of Russia, 2007–2023.

Variable Factors	Logistic Regression Coefficient	OR	OR 95% CI	Std. Error	*p*-Value
Intercept	0.327	1.38	0.287–3.491	0.137	0.000
Wild boar population density	1.258	3.51	2.983–130.37	0.089	0.000
Carcasses of wild boar	0.544	1.72	1.562–5.324	0.052	0.000
ASF outbreaks in pigs	0.066	1.07	1.032–4.105	0.017	0.000
Forestry areas	0.001	1.05	1.000–2.303	0.001	0.006

Footnote: OR—odds ratio, CI—confidence interval.

**Table 3:** Statistical metrics of the general logistic regression model to determine risk factors of ASFV among the wild boar population in the Far East of Russia, 2007–2023.

Variable Factors	Logistic Regression Coefficient	OR	OR 95% CI	Std. Error	*p*-Value
Intercept	1.183	3.26	1.654–23.387	0.453	0.000
Number of hunting farms	4.044	57.05	3.845–185.345	1.362	0.000
Wild boar population density	3.564	35.30	2.082–153.069	1.904	0.011
Carcasses of wild boar	0.322	1.38	1.237–14.575	0.062	0.000
Number of settlements	0.212	1.24	1.009–3.016	0.002	0.000

Footnote: OR—odds ratio, CI—confidence interval.

**Table 4:** Relative importance of variables based on random forest analysis results in the European part of Russia, 2007–2023.

Variable Factors	Mean Decrease Gini	Mean Decrease Accuracy
Number of hunting farms	105.73	84.22
Wild boar population density	62.36	50.72
Carcasses of wild boar	29.07	18.67
Forestry areas	21.58	4.14
Number of settlements	18.23	1.34
ASF outbreaks in pigs	16.89	19.32

**Table 5:** Relative importance of variables based on random forest-based classification analysis results in the Far East in Russia, 2007–2023.

Variable Factors	Mean Decrease Gini	Mean Decrease Accuracy
Wild boar population density	140.67	97.80
Carcasses of wild boar	57.19	17.24
Forestry areas	40.99	3.43
Number of settlements	38.80	2.36
ASF outbreaks in pigs	23.79	34.18

**Table 6:** Characteristics of performance metrics of logistic and random forest regression models.

Type of Regression Models	Dataset	R^2^	AUC	Accuracy
Generalized Linear Logistic Regression Model (GLLRM)	Training I	0.67	0.84	0.81
	Testing I	0.65	0.78	0.87
	Training II	0.70	0.76	0.84
	Testing II	0.68	0.75	0.88
Random Forest Model (RFM)	Training I	0.80	0.96	0.96
	Testing I	0.78	0.94	0.91
	Training II	0.75	0.86	0.98
	Testing II	0.74	0.81	0.94

Footnote: Dataset Training I and Dataset Testing I—regression model of European part and Dataset Training II and Dataset Testing II—regression model of Far Eastern part of the Russian Federation.

## Data Availability

The data presented in this study are available upon request from the corresponding author.

## References

[1] Chenais E., Ståhl K., Guberti V., Depner K. (2018). Identification of wild boar-habitat epidemiologic cycle in African swine fever epizootic. Emerg. Infect. Dis..

[2] Nielsen S.S., Alvarez J., Bicout D.J., Calistri P., Depner K., Drewe J.A., Garin-Bastuji B., Rojas J.L.G., Schmidt C.G., European Food Safety Authority (EFSA) (2021). ASF Exit Strategy: Providing cumulative evidence of the absence of African swine fever virus circulation in wild boar populations using standard surveillance measures. EFSA J..

[3] Gallardo M.C., de la Torre Reoyo A., Fernández-Pinero J., Iglesias I., Muñoz M.J., Arias M.L. (2015). African swine fever: A global view of the current challenge. Porc. Health Manag..

[4] Guinat C., Gogin A., Blome S., Keil G., Pollin R., Pfeiffer D.U., Dixon L. (2016). Transmission routes of African swine fever virus to domestic pigs: Current knowledge and future research directions. Vet. Rec..

[5] Bergmann H., Schulz K., Conraths F.J., Sauter-Louis C. (2021). A review of environmental risk factors for african swine fever in european wild boar. Animals.

[6] Boklund A., Dhollander S., Chesnoiu Vasile T., Abrahantes J.C., Bøtner A., Gogin A., Gonzalez Villeta L.C., Gortázar C., More S.J., Papanikolaou A. (2020). Risk factors for African swine fever incursion in Romanian domestic farms during 2019. Sci. Rep..

[7] Gervasi V., Marcon A., Guberti V. (2022). Estimating the risk of environmental contamination by forest users in African Swine Fever endemic areas. Acta Vet. Scand..

[8] Gervasi V., Guberti V. (2021). African swine fever endemic persistence in wild boar populations: Key mechanisms explored through modelling. Transbound. Emerg. Dis..

[9] Nielsen S.S., Alvarez J., Bicout D.J., Calistri P., Depner K., Drewe J.A., Garin-Bastuji B., Gonzales Rojas J.L., Schmidt C., Herskin M. (2021). Research objectives to fill knowledge gaps in African swine fever virus survival in the environment and carcasses, which could improve the control of African swine fever virus in wild boar populations. EFSA J..

[10] Pepin K.M., Borowik T., Frant M., Plis K., Podgórski T. (2023). Risk of African swine fever virus transmission among wild boar and domestic pigs in Poland. Front. Vet. Sci..

[11] Pepin K.M., Golnar A.J., Abdo Z., Podgórski T. (2020). Ecological drivers of African swine fever virus persistence in wild boar populations: Insight for control. Ecol. Evol..

[12] Zakharova O.I., Korennoy F.I., Yashin I.V., Burova O.A., Liskova E.A., Gladkova N.A., Razheva I.V., Blokhin A.A. (2023). Spatiotemporal Patterns of African Swine Fever in Wild Boar in the Russian Federation (2007–2022): Using Clustering Tools for Revealing High-Risk Areas. Animals.

[13] Zakharova O.I., Blokhin A.A., Toropova N.N., Burova O.A., Yashin I.V., Korennoy F.I. (2022). Density of wild boar population and spread of African swine fever in the Russian Federation. Vet. Sci. Today.

[14] Schulz K., Staubach C., Blome S., Viltrop A., Nurmoja I., Conraths F.J., Sauter-Louis C. (2019). Analysis of Estonian surveillance in wild boar suggests a decline in the incidence of African swine fever. Sci. Rep..

[15] De Vos C.J., Taylor R.A., Simons R.R.L., Roberts H., Hultén C., De Koeijer A.A., Lyytikäinen T., Napp S., Boklund A., Petie R. (2019). Generic approaches for Risk Assessment of Infectious animal Disease introduction (G-RAID). EFSA Support. Publ..

[16] EFSA Panel on Animal Health and Welfare (AHAW) (2012). Guidance on Risk Assessment for Animal Welfare. EFSA J..

[17] Lim J.S., Andraud M., Kim E., Vergne T. (2023). Three Years of African Swine Fever in South Korea (2019–2021): A Scoping Review of Epidemiological Understanding. Transbound. Emerg. Dis..

[18] Zakharova O.I., Blokhin A.A., Yashin I.V., Kolbasov D.V., Korennoy F.I., Burova O.A. (2023). Investigation of Risk Factors Associated with the African Swine Fever Outbreaks in the Nizhny Novgorod Region of Russia, 2011–2022. Transbound. Emerg. Dis..

[19] Cappai S., Rolesu S., Coccollone A., Laddomada A., Loi F. (2018). Evaluation of biological and socio-economic factors related to persistence of African swine fever in Sardinia. Prev. Vet. Med..

[20] Zakharova O.I., Titov I.A., Gogin A.E., Sevskikh T.A., Korennoy F.I., Kolbasov D.V., Abrahamyan L., Blokhin A.A. (2021). African Swine Fever in the Russian Far East (2019–2020): Spatio-Temporal Analysis and Implications for Wild Ungulates. Front. Vet. Sci..

[21] Masiulis M., Bušauskas P., Jonušaitis V., Pridotkas G. (2019). Potential role of domestic pig carcasses disposed in the forest for the transmission of African swine fever. Berl. Munch. Tierarztl. Wochenschr..

[22] Zani L., Masiulis M., Bušauskas P., Dietze K., Pridotkas G., Globig A., Blome S., Mettenleiter T., Depner K., Karvelienė B. (2020). African swine fever virus survival in buried wild boar carcasses. Transbound. Emerg. Dis..

[23] WAHIS. https://wahis.woah.org/#/event-management.

[24] Bellini S., Casadei G., De Lorenzi G., Tamba M. (2021). A Review of Risk Factors of African Swine Fever Incursion in Pig Farming within the European Union Scenario. Pathogens.

[25] Blagus R., Lusa L. (2013). SMOTE for high-dimensional class-imbalanced data. BMC Bioinform..

[26] Chawla N.V., Bowyer K.W., Hall L.O., Kegelmeyer W.P. (2002). SMOTE: Synthetic minority over-sampling technique. J. Artif. Intell. Res..

[27] Weisberg S., Price B., Friendly M., Firth D., Taylor S. (2022). Package ‘Effects’. https://cran.r-project.org/web/packages/car/car.pdf.

[28] Bangdiwala S.I. (2018). Regression: Binary logistic. Int. J. Inj. Control Saf. Promot..

[29] Sroka C.J., Nagaraja H.N. (2018). Odds ratios from logistic, geometric, Poisson, and negative binomial regression models. BMC Med. Res. Methodol..

[30] van Smeden M., Moons K.G.M., de Groot J.A.H., Collins G.S., Altman D.G., Eijkemans M.J.C., Reitsma J.B. (2019). Sample size for binary logistic prediction models: Beyond events per variable criteria. Stat. Methods Med. Res..

[31] Loh W.Y. (2011). Classification and regression trees. Wiley Interdiscip. Rev. Data Min. Knowl. Discov..

[32] Breiman L., Friedman J.H., Olshen R.A., Stone C.J. (2017). Classification and Regression Trees.

[33] Amaral P.V., Anselin L. (2014). Finite sample properties of Moran’s I test for spatial autocorrelation in tobit models. Pap. Reg. Sci..

[34] Arlot S., Celisse A. (2010). A survey of cross-validation procedures for model selection. Stat. Surv..

[35] Bates S., Hastie T., Tibshirani R. (2024). Cross-Validation: What Does It Estimate and How Well Does It Do It?. J. Am. Stat. Assoc..

[36] Catalano M., Leise T., Pfaff T. (2009). Measuring Resource Inequality: The Gini Coefficient. Numeracy.

[37] Jaiswal J.K., Samikannu R. Application of Random Forest Algorithm on Feature Subset Selection and Classification and Regression. Proceedings of the 2017 World Congress on Computing and Communication Technologies (WCCCT).

[38] Boklund A.E., Ståhl K., Miranda Chueca M.Á., Podgórski T., Vergne T., Cortiñas Abrahantes J., Cattaneo E., Dhollander S., Papanikolaou A., Tampach S. (2024). Risk and protective factors for ASF in domestic pigs and wild boar in the EU, and mitigation measures for managing the disease in wild boar. EFSA J..

[39] Guinat C., Vergne T., Jurado-Diaz C., Sánchez-Vizcaíno J.M., Dixon L., Pfeiffer D.U. (2017). Effectiveness and practicality of control strategies for African swine fever: What do we really know?. Vet. Rec..

[40] Taylor R.A., Condoleo R., Simons R.R.L., Gale P., Kelly L.A., Snary E.L. (2020). The Risk of Infection by African Swine Fever Virus in European Swine Through Boar Movement and Legal Trade of Pigs and Pig Meat. Front. Vet. Sci..

[41] Baños J.V., Boklund A., Gogin A., Gortázar C., Guberti V., Helyes G., Kantere M., Korytarova D., Linden A., Masiulis M. (2022). Epidemiological analyses of African swine fever in the European Union. EFSA J..

[42] Podgórski T., Śmietanka K. (2018). Do wild boar movements drive the spread of African Swine Fever?. Transbound. Emerg. Dis..

[43] Cavazza S., Brogi R., Apollonio M. (2024). Sex-specific seasonal variations of wild boar distance traveled and home range size. Curr. Zool..

[44] Goicolea T., Cisneros-araújo P., Sánchez-vizcaíno J.M., Bosch J. (2024). Landscape connectivity for predicting the spread of ASF in the European wild boar population. Sci. Rep..

[45] Jiang D., Ma T., Hao M., Ding F., Sun K., Wang Q., Kang T., Wang D., Zhao S., Li M. (2022). Quantifying risk factors and potential geographic extent of African swine fever across the world. PLoS ONE.

[46] Guberti V., Khomenko S., Masiulis M., Kerba S. (2022). African Swine Fever in Wild Boar Ecology and Biosecurity.

[47] Lange M., Guberti V., Thulke H. (2018). Understanding ASF spread and emergency control concepts in wild boar populations using individual-based modelling and spatio-temporal surveillance data. EFSA Support. Publ..

[48] Glazunova A.A., Korennoy F.I., Sevskikh T.A., Lunina D.A., Zakharova O.I., Blokhin A.A., Karaulov A.K., Gogin A.E. (2021). Risk Factors of African Swine Fever in Domestic Pigs of the Samara Region, Russian Federation. Front. Vet. Sci..

[49] Loh W.-Y. (2012). Variable Selection for Classification and Regression in Large p, Small n Problems. Probability Approximations and Beyond.

[50] (2023). Methodology for Assessing the Number of Hunting Resources Using the Winter Route Method.

[51] Accounting Methods for 2023. http://www.ohotcontrol.ru/materials/metodiki-2022/.

[52] Wijeyakulasuriya D.A., Eisenhauer E.W., Shaby B.A., Hanks E.M. (2020). Machine learning for modeling animal movement. PLoS ONE.

[53] Altizer S., Bartel R., Han B.A. (2011). Animal migration and infectious disease risk. Science.

